# Peripartum hysterectomy: prevalence, indications, maternal outcomes, and associated factors in a 7-year retrospective review at Wolaita Sodo University Comprehensive Specialized Hospital, Southern Ethiopia

**DOI:** 10.1186/s12884-025-07616-x

**Published:** 2025-04-24

**Authors:** Habtamu Desalegn, Amanuel Geta, Debritu Nane

**Affiliations:** 1 College of Health Science and Medicine, School of Medicine, Department of Obstetrics and Gynecology, Wolaita Sodo University, Wolaita Sodo, Ethiopia; 2 College of Health Science and Medicine, School of Public Health, Department of Reproductive Health and Nutrition, Wolaita Sodo University, Wolaita Sodo, Ethiopia

**Keywords:** Peripartum hysterectomy, Prevalence, Indications, Maternal outcomes, Factors, Ethiopia

## Abstract

**Background:**

Peripartum hysterectomy (PH) is a life-saving surgical procedure for severe childbirth complications such as uncontrollable bleeding, uterine rupture, or severe infection. Despite its crucial role in obstetric care, research on the prevalence of PH and the factors contributing to its necessity remains limited, necessitating further evaluation.

**Objective:**

To evaluate the the prevalence, indications, and maternal outcomes of PH and identifying factors associated with maternal outcomes among women who underwent PH at Wolaita Sodo University Comprehensive Specialized Hospital, Southern Ethiopia.

**Methods:**

A retrospective cross-sectional study was conducted on 162 participants using data from March 2017 to April 2023. Data were extracted from patient records between February and April 2023 and analyzed using SPSS version 26. Descriptive statistics were reported, and variables with *p* < 0.25 in bivariate analysis were included in the multivariable model, with statistical significance set at *p* ≤ 0.05.

**Results:**

A review of 162 maternal charts involving PH showed a rate of 5.2 per 1,000 deliveries. Most hysterectomies (87%) were performed following cesarean delivery. The primary indications for PH included uterine rupture (75.3%), uterine atony (17.3%), placenta accreta (4.9%), and deep surgical site infection (1.9%). Severe maternal morbidity included wound infections (24.7%), adjacent structure injuries (8.6%), re-laparotomies (7.4%), and other complications (6.8%). Intensive care unit admission occurred in 37%, and 83.3% required blood transfusions. Maternal and perinatal mortality rates were 23.5% and 75.9%, respectively. The key predictors of maternal outcome included delivery in the study hospital, pre-operative blood pressure of < 90/60 mmHg, and pre-operative blood transfusion.

**Conclusion:**

PH is a major contributor to maternal morbidity and mortality, primarily due to uterine rupture. The relatively high PH rate suggests significant maternal health challenges. To address this, improvements in preoperative care, early detection of complications, and enhanced emergency obstetric services could help reduce the need for hysterectomy and improve maternal and perinatal outcomes.

## Introduction

Peripartum hysterectomy (PH) is a ‟near miss” maternal event- an intervention performed in life-threatening obstetric hemorrhage to prevent maternal death. It results in the loss of fertility and is associated with significant maternal complications [1]. Intra-operative and post-operative complications associated with PH include febrile morbidity, bladder injury, re-exploration, wound dehiscence, extended hospital stays over 7 days, blood transfusions exceeding 2 units, disseminated intravascular coagulation, as well as maternal and fetal death [2]. The mortality and morbidity associated with performing a PH are significantly higher, particularly in critically ill patients referred from other Hospitals [2].

Globally, the incidence of PH varies widely. In high-income countries, the rate of PH is relatively low, affecting less than 1 in 1,000 deliveries [3]. In United States, the incidence rates of PH ranged from 0.8 per 1,000 deliveries in 1987 to 2.28 per 1,000 deliveries in 2006 [4]. However, in low- and middle-income countries (LMICs), the burden is considerably higher due to limited access to quality obstetric care, delayed referrals, and higher rates of obstetric complications. In Nigeria and Pakistan, the incidence of PH is reported to be 4 and 11 per 1,000 deliveries, respectively [3].

In Ethiopia, the prevalence of PH varies across different studies and regions. A retrospective review conducted at St. Paul’s Hospital Millennium Medical College in Addis Ababa reported an incidence of 2.6 per 1,000 deliveries [5].

The risk factors for PH increase with increasing maternal age, increasing parity, abnormal placentation, and cesarean delivery in previous and current pregnancies [6]. Inadequate infrastructure and delayed referrals significantly contribute to the rising trend of obstetric hysterectomy [7]. Several studies reported placental pathologies and the cesarean sections as risk factors for PH [8–10].

Indications for PH have changed throughout the years. In the past, the major indications for emergency PH were uterine rupture and atony. However, recent studies have listed abnormal placentation as the leading cause of PH. The increase in the cesarean section (CS) rate is the leading cause to an increase in the rate of abnormal placentation, which in turn gives rise to an increase in PH rate [11].

Emergency peripartum hysterectomy (EPH), is defined as an emergency hysterectomy within 6 weeks of post-partum [12]. EPH is a procedure performed at the time of delivery or in the postpartum period as a life-saving measure in response to severe post-partum hemorrhage that does not respond to any other interventions. It can follow a cesarean section or a vaginal delivery [13]. The rate of EPH is more common in developing countries like Ethiopia due to high incidence of unbooked and improperly supervised deliveries outside the hospitals and lack of skilled health professionals [14].

The complication associated with EPH is significant in our country as well as in our hospital. Despite the clinical significance of PH, there is a notable paucity of research on its prevalence, indications, outcomes, and associated risk factors in Southern Ethiopia. Most existing studies in Ethiopia are confined to a few tertiary hospitals in other regions, and local data from Wolaita Sodo University Comprehensive Specialized Hospital are lacking. Understanding the burden and clinical profile of PH in this setting is critical to informing evidence-based interventions aimed at improving maternal outcomes.

Therefore, this study aims to assess the prevalence, indications, maternal outcomes, and associated factors of peripartum hysterectomy over a seven-year period at Wolaita Sodo University Comprehensive Specialized Hospital. The findings will contribute valuable evidence to inform health care planning, emergency obstetric care training, and quality improvement initiatives tailored to reduce preventable maternal morbidity and mortality in Southern Ethiopia.

## Methods and materials

### Study settings

The study was conducted at Wolaita Sodo University Comprehensive Specialized Hospital (WSUCSH), located in the Southern Ethiopia Region, 380 km away from Addis Ababa, the capital of Ethiopia. The Hospital is located in Sodo town, within the Wolaita Zone, and serves a catchment area with a population of around two million, including residents from neighboring zones. On average, the outpatient department receives about 40 pregnant women daily for antenatal care (ANC). The Hospital has more than 437 beds, with 60 of these allocated to the obstetrics and gynecology ward, 10 beds in the labor ward, and 50 in the gynecology and maternity ward. The obstetrics and gynecology department has its own building, which includes a neonatal care unit, outpatient department, emergency outpatient department, ANC, family planning, Expanded Program of Immunization, labor and delivery unit, postnatal care unit, maternity ward, gynecology ward, operation theater, post-anesthesia care unit, recovery unit, pharmacy, and laboratory services for mothers and neonates. The department is staffed by 11 gynecologists, 62 midwives (BSc and diploma holders), 39 residents, 17 general practitioners, and medical interns.

### Study design, period and population

This retrospective cross-sectional study analyzed records from March 2017 to April 2023, with data extraction carried out between February 1 and April 30, 2023. A total of 30,956 patient records were initially reviewed. Of these, there were 178 women who underwent PH. Subsequently, 16 cases were excluded due to incomplete data, resulting in a final sample of 162 participants included in the study. All pregnant mothers who gave birth whether by cesarean section, instrumental delivery or spontaneous vaginal delivery and visited WSUCSH were the source population. The study population included all mothers who underwent PH within 6 weeks of delivery at WSUCSH over the seven-year study period.

### Sampling method and technique

A total population sampling technique was employed, where all eligible medical records from March 2017 to April 2023 were included in the analysis.

### Data quality control

Before collection of data, orientation was given to data collectors and supervisors about the objective of the study and data collection tools. Data was collected by two midwives and one general practitioner. One resident physician was assigned as supervisor. To ensure quality data collection, close supervision was carried out by principal investigator during data abstraction. Data abstraction form was checked for completeness and consistency.

## Study variables

### Dependent variables

- Peripartum hysterectomy.
- Maternal outcome (Alive/Died).
- Neonatal outcome (Alive/Died).

### Independent variables

- Age.
- Residence.
- Educational status.
- Source of referral.
- Parity.
- Gestational age.
- Previous CS.
- Place of delivery.
- Mode of delivery.
- ANC contact.
- Uterine rupture.
- Uterine atony.
- Placenta previa.

### Operational and term definitions

#### Peripartum hysterectomy

A surgical removal of the uterus performed during delivery or within six weeks of postpartum [15].

#### Febrile morbidity

A body temperature of 38 °C or higher measured at least 24 h after hysterectomy and repeated at least once.

#### Operative vaginal delivery

Delivery in which practitioner uses either vacuum or forceps with or without the assistance of maternal pushing [16].

#### Primiparity

Given birth to a child once after viability [17].

#### Multiparity

Given birth of two to five children [18].

#### Grandmultiparity

A woman who has experienced five or more previous deliveries beyond the second trimester of pregnancy [19].

#### Puerperal sepsis

It is an infection that occurs between labor and 42 days post-partum with a fever and at least one of the following symptoms: pelvic pain, abnormal vaginal discharge, or delay in uterine involution [20].

#### Post-partum hemorrhage

Excessive bleeding following childbirth that leads to clinical signs and symptoms of hypovolemia, such as low blood pressure, rapid pulse, dizziness, or pallor [15].

#### Wound infection

The presence of any two of the following: purulent discharge and/or obvious cellulitis, elevated temperature, and positive wound culture [21].

### Data processing and analysis

Data was coded, checked, and entered in to EpiData version 3.1 software, and exported to SPSS version 26 for analysis. Descriptive analysis was carried out and presented by using frequencies, percentage and measure of central tendency and dispersion. Binary logistic regression models were computed for both bi-variate and multi-variable analysis to identify predictors. Those variables that have p value less than 0.25 in bi-variable binary logistic regression models were considered as candidates for the multi-variable logistic regression. P value less than 0.05 was considered as statistically significant. Both crude and adjusted odds ratio with the respective 95% confidence was computed to show strength of association. In this analysis, the dependent variable is binary, there is no dependence between subjects, and multicollinearity among predictors was assessed using the variance inflation factor (VIF), with all variables showing acceptable correlation (VIF < 10).

## Results

### Socio-demographic characteristics of participants

A total of 30,956 deliveries were registered, of which 162 participants underwent PH. The mean age of the patients was 30 years, with a range of 17 to 40 years. Among the participants, 140 (86.4%) were from rural areas, while 22 (13.6%) were from urban areas. Regarding parity, 54% were multiparous, 38.3% were grand multiparous, and a smaller proportion was nulliparous or primiparous. In terms of gestational age, the majority (88.9%) had an unknown gestational age, 5.6% had term pregnancies, 4.9% had preterm pregnancies, and 0.6% had post-term pregnancies. Regarding antenatal care (ANC), 113 (70.6%) participants attended ANC visits, while 38.2% did not. In terms of educational level, 103 (63.6%) participants were illiterate, indicating a high proportion of women without formal education.

### Distribution of obstetric complications by socio-demographic and clinical characteristics among participants

Obstetric characteristics reveal that 96.3% of the peripartum hysterectomies were performed within 24 h of delivery, with 87.0% of the procedures following cesarean delivery (CD) and 13.0% following vaginal delivery (VD). The most common indication for EPH was uterine rupture, which occurred in 71.6% of CD cases and 3.7% of VD cases. Regarding previous cesarean deliveries, 129 (79.6%) participants had no history of CD, and among these, 99 (61.1%) experienced uterine rupture. When considering the indications for EPH by residence, uterine rupture was the leading cause in both rural and urban areas. In rural areas, 115 (71.0%) cases were attributed to uterine rupture, followed by uterine atony (19, 11.7%), placenta accreta (4, 2.5%), placenta previa (1, 0.6%), and deep surgical site infection (SSI) (1, 0.6%). In urban areas, uterine rupture occurred in 7 cases (4.3%), uterine atony in 9 cases (5.6%), placenta accreta in 4 cases (2.5%), and deep SSI in 2 cases (1.2%).

By ANC attendance, among those who attended ANC (113 participants), uterine rupture was the most common indication 79 (48.8%), followed by uterine atony 23 (14.2%), placenta accreta 8 (4.9%), and deep SSI 3 (1.9%). In contrast, for participants who did not attend ANC (49 participants), uterine rupture was still the most common cause 43 (26.5%), with fewer cases of uterine atony 5 (3.1%) and placenta previa 1 (0.6%).

Ethnicity also played a role in the indications for EPH. Among Wolaita ethnic participants (115), uterine rupture occurred in 79 cases (48.8%), followed by uterine atony in 24 (14.8%), placenta accreta in 8 (4.9%), placenta previa in 1 (0.6%), and deep SSI in 3 (1.9%). In patients from other ethnicities (47 participants), uterine rupture was the dominant indication (43, 26.5%), followed by uterine atony in 4 (2.5%).

Educational level was linked to the indications for peripartum hysterectomy as well. Among illiterate participants (103), uterine rupture was the most common cause (86, 53.1%), with smaller proportions of other indications. In multiparous women (54%), uterine rupture occurred in 61 cases (37.7%), reflecting the high burden of this complication in women with multiple previous pregnancies. Finally, for those without a history of cesarean delivery (129 participants), uterine rupture was again the most frequent indication, affecting 99 (61.1%) of these women. (Table 1)

**Table 1:** Patient and obstetric characteristics of cases of PH by indication at WSUCHS from 2017–2023

Variables		Uterine rupture *n* (%)	Uterine atony *n* (%)	Placenta accreta *n* (%)	Placenta previa *n* (%)	Deep SSI *n* (%)	Total *n* (%)
Residence	Rural	115 (71.0)	19 (11.7)	4 (2.5)	1 (0.6)	1 (0.6)	140 (86.4)
	Urban	7 (4.3)	9 (5.6)	4 (2.5)	0 (0.0)	2 (1.2)	22 (13.6)
ANC visit	Yes	79 (48.8)	23 (14.2)	8 (4.9)	0 (0.0)	3 (1.9)	113 (70.6)
	No	43 (26.5)	5 (3.1)	0 (0.0)	1 (0.6)	0 (0.0)	49 (29.4)
Ethnicity	Wolaita	79 (48.8)	24 (14.8)	8 (4.9)	1 (0.6)	3 (1.9)	115 (71.0)
	Others	43 (26.5)	4 (2.5)	0 (0.0)	0 (0.0)	0 (0.0)	47 (29)
Timing of EPH	After CD	116 (71.6)	13 (8.0)	8 (4.9)	1 (0.6)	3 (1.9)	141 (87.0)
	After VD	6 (3.7)	15 (9.3)	0 (0.0)	0 (0.0)	0 90.0)	21 (13.0)
Education	Illiterate	86 (53.1)	13 (8.0)	2 (1.2)	1 (0.6)	1 (0.6)	103(63.6)
	Primary	32 (19.8)	8 (4.9)	4 (2.5)	0 (0.0)	0 (0.0)	44 (27.1)
	Secondary	4 (2.5)	5 (3.1)	1 (0.6)	0 (0.0)	1 (0.6)	11 (6.8)
	College and above	0 (0.0)	2 (1.2)	1 (0.6)	0 (0.0)	1 (0.6)	4 (2.5)
Parity	Primi-parous	7 (4.3)	0 (0.0)	0 (0.0)	0 (0.0)	1 (0.6)	8 (4.9)
	Multi-parous	61 (37.7)	19 (11.7)	7 (4.3)	1 (0.6)	1 (0.6)	89 (54.9)
	Grand multiparous	52 (32.1)	8 (4.9)	1 (0.6)	0 (0.0)	1 (0.6)	62 (38.3)
	Nulliparous	2 (1.2)	1 (0.6)	0 (0.0)	0 (0.0)	0 (0.0)	3 (1.9)
Previous CD	Yes	23 (14.2)	3 (1.9)	7 (4.3)	0 (0.0)	0 (0.0)	33 (20.4)
	No	99 (61.1)	25 (15.4)	1 (0.6)	1 (0.6)	3 (1.9)	129 (79.6)
GA	Preterm	5 (3.1)	2 (1.2)	1 (0.6)	0 (0.0)	0 (0.0)	8 (4.9)
	Unknown	112 (69.1)	23 (14.2)	5 (3.1)	1 (0.6)	3 (1.9)	144 (88.9)
	Term	4 (2.5)	3 (1.9)	2 (1.2)	0 (0.0)	0 (0.0)	9 (5.6)
	Post term	1 (0.6)	0 (0.0)	0 (0.0)	0 (0.0)	0 (0.0)	1 (0.6)

SSI, surgical site infection; ANC, antenatal care; EPH, emergency peripartum hysterectomy; VD, vaginal delivery; CD, cesarean delivery; GA, gestational age

### Indications for PH

The most common indication for PH was uterine rupture, accounting for 122 out of 162 cases (75.3%). Among these, 116 occurred during CD, three followed spontaneous vertex delivery, and the remaining three were associated with operative vaginal delivery. Of the 122 uterine rupture cases, 115 were referred from primary hospitals and health centers, with 52 of these cases involving grand multiparous women. Additionally, 79 of the 122 women had attended ANC, while 43 had not.

The second most common indication for PH was uterine atony, with 28 cases reported. In all cases of uterine atony, conservative methods were attempted before proceeding to hysterectomy. Specifically, medical conservative methods were tried in 23 cases, while in 2 cases, a surgical approach was directly implemented due to the patients’ deteriorating conditions, and both surgical and medical methods were applied in the remaining 3 cases. The third indication was placenta accreta syndrome, with eight cases identified, seven of which involved a history of cesarean delivery, and all cases had a cesarean scar of two or more. Other indications included deep surgical site infections (*n* = 3) and placenta previa (*n* = 1).

A total hysterectomy was performed in 138 out of 162 cases (85.2%), with uterine rupture being the most frequent indication, accounting for 119 of these total hysterectomies. The remaining 24 patients (14.8%) underwent subtotal hysterectomy, most commonly due to uterine atony (19 out of 24 cases). All hysterectomies were performed by either a senior obstetrics resident or a consultant obstetrician (Table 2).

**Table 2:** Indications for PH at WSUCSH from 2017–2023

Indications for PH	Number of deaths (*n*)	Percentage (%)
Uterine rupture	122	75.3
Uterine atony	28	17.3
Placenta accreta	8	4.9
Deep SSI	3	1.9
Placenta previa	1	0.6
Total	38	100

### SSI, surgical site infection

#### Neonatal outcome of PH

**Fig. 1:**
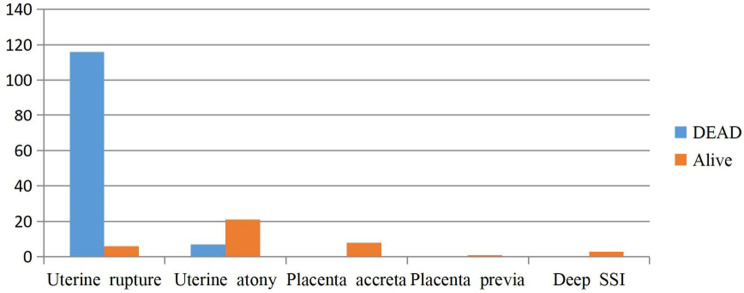
Neonatal outcome with indications of PH at WSUCSH from 2017–2023. This bar graph illustrates the distribution of maternal outcomes based on different obstetric complications, with outcomes categorized as “Dead” (blue bars) and “Alive” (red bars). The complications shown include uterine rupture, uterine atony, placenta accreta, placenta previa, and deep surgical site infection (SSI)

Perinatal mortality was 123 out of 162 cases (75.9%), with the majority of stillbirths occurring in women diagnosed with uterine rupture (Fig. 1).

### Maternal outcome of PH

**Fig. 2:**
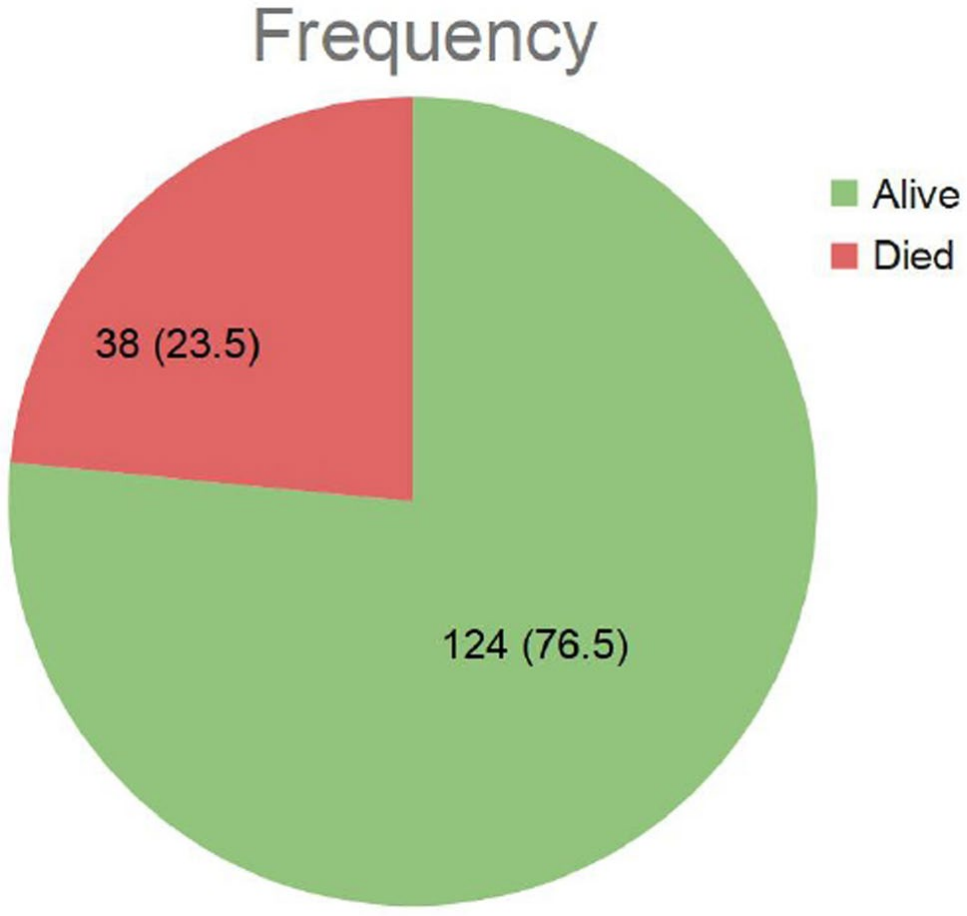
Maternal outcomes among women who underwent PH. This pie chart presents the distribution of maternal outcomes among 162 women who underwent PH. Of the total cases, 124 women (76.5%) survived, while 38 women (23.5%) died, resulting in an overall maternal mortality rate of 23.5%

The overall maternal mortality rate was 38 out of 162 cases (23.5%) (Fig. 2).

The primary cause of maternal death was multiple organ failure (MOF) secondary to shock, which accounted for 31 out of the 38 deaths. This was followed by pulmonary thromboembolism (2 out of 38), cardiac arrest (2 out of 38), and amniotic fluid embolism (AFE) (2 out of 38). Additionally, one mother died due to disseminated intravascular coagulation (DIC). The specific causes of maternal death are detailed in Table 3.

**Table 3:** Causes of death in mothers undergone PH at WSUCSH from 2017–2023, (*n* = 38)

Cause of maternal death	Number of deaths (*n*)	Percentage (%)
MOF secondary to shock	31	81.6
Pulmonary thromboembolism	2	5.3
Cardiac arrest	2	5.3
AFE	2	5.3
DIC	1	2.6
Total	38	100

MOF, Multiple organ failure; AFE, Amniotic fluid embolism; DIC, Disseminated intravascular coagulation

### Maternal complications associated with PH

Mothers who survived the operation also experienced complications during or after surgery, with 77 out of 124 (62.1%) affected. The most common complication was surgical site infection, occurring in 40 (24.7%) of cases. Other complications included injury to adjacent structures 14 (8.6%), re-laparotomy 12 (7.4%), and other issues 11 (6.8%) (Table 4).

**Table 4:** Complications associated with PH among mothers who survived at WSUCSH from 2017 to 2023 (*n* = 124)

Complication	Number (*n*)	Percentage (%)
**Total complications**	77	62.1
Surgical site infection	40	24.7
Injury to adjacent structures	14	8.6
Relaparotomy	12	7.4
Other complications	11	6.8

### Intensive care unit (ICU) admissions, ward stays, and blood transfusion requirements among post-operative patients

The rate of ICU admission was 60 out of 162 cases (37%). Among those admitted to the ICU, 24 out of 60 (14.8%) stayed in the ICU for two to seven days, 35 out of 60 (21.6%) stayed for two days or less, and one woman remained in the ICU for more than a week. Of the patients admitted to the ward, 89 out of 127 (54.9%) stayed in the ward for a week or more. Blood transfusion was required in 83.3% of the cases, with 80.9% of those needing 1 to 5 units of blood products, while the remaining 2.5% required 6 to 10 units of blood products (Table 5).

**Table 5:** ICU admissions, ward stays, and blood transfusion requirements among mothers who survived surgery at WSUCSH from 2017–2023

Catagory	Frequency	Percent
**ICU Admissions (** ***n*** ** = 60)**		
ICU stay (2–7 days)	24	14.8
ICU stay (2 days or less)	35	21.6
ICU stay (more than 7 days)	1	0.6
**Ward Admissions (** ***n*** ** = 127)**		
Ward stay (1 week or more)	89	54.9
**Blood Transfusions (** ***n*** ** = 135)**		
1 to 5 units of blood products	131	80.9
6 to 10 units of blood products	4	2.5

ICU, intensive care unit

### Factors associated with PH and maternal outcome

Crude association of each variable on maternal outcome after EPH was checked. The maternal factor variables; age, history of cesarean section, history of APH, blood pressure before surgery, delivery in the study hospital, number of babies delivered, and transfusion with blood products were candidates for multivariate analysis with p value less than 0.25 on crude association.

Independent variables with a p-value less than 0.25 in the bivariate logistic regression were included in the final multivariable logistic regression analysis. In the multivariable model, maternal blood pressure before surgery was significantly associated with maternal survival. Women with the preoperative blood pressure of < 90/60 mmHg had 68% reduced odds of survival (AOR = 0.32; 95% CI: 0.1–0.8) compared with their counterparts. Receiving a pre-operative blood transfusion had 6.8 times higher odds of survival (AOR = 6.8; 95% CI: 1.1 − 43.4). Additionally, delivery within the study hospital was significantly associated with increased maternal survival (AOR = 16.1; 95% CI: 2.2–117.7) **(**Table 6**).**

**Table 6:** Bivariate and multi variable analysis of maternal outcome after PH at WSUCSH from 2017–2023

Variables		Maternal survival		COR (95% CI)	AOR (95% CI)	P-value
		Yes	No			
Age (years)	≤ 30	88	20	2.2 (1.0–4.6)	2.2 (0.9–5.3)	0.08
	> 30	36	18	1	1	
Mode of delivery	SVD	9	5	0.5 (0.2–1.6)	0.8 (0.2–3.5)	0.7
	OVD	4	3	0.4 (0.8–1.7)	1.3 (0.8–8.7)	
	CD	111	30	1	1	
History of CS	Yes	29	4	2.59 (0.42–1.83)	2.4 (0.7–8.4)	0.2
	No	95	34	1	1	
History of APH	Yes	32	16	0.5 (0.2–1.0)	0.4 (0.1–1.2)	0.09
	No	92	22	1	1	
BP before surgery (mmHg)	< 90/60	38	24	0.3 (0.1–0.6)	0.3 (0.1–0.8)*	0.01
	≥ 90/60	86	14	1	1	
Number of babies delivered	Singleton	111	37	0.2 (0.1–1.8)	0.1 (0.01–1.2)	0.06
	Twin	13	1	1	1	
Transfused with blood products	Yes	99	36	4.5 (1.01–20.2)	6.8 (1.1–43.4)*	0.04
	No	25	2	1	1	
Delivery in study hospital	Yes	121	32	7.6 (1.8–31.9)	16.1 (2.2–117.7)*	0.006
	No	3	6	1	1	

SVD; spontaneous vaginal delivery, CD; Cesearean delivery, CS; cesarean section, APH; antepartum haemorrhage, BP; blood pressure

## Discussion

This study aimed to assess the prevalence, indications, maternal outcomes, and associated factors at WSUCSH. The findings highlighted that most PH procedures were performed following cesarean deliveries, with uterine rupture being the leading indication. Key predictors of maternal outcomes included delivery at the study hospital, low pre-operative blood pressure, and pre-operative blood transfusion.

The present study, with an incidence of 5.2 per 1000 deliveries, reports the highest rate seen. In contrast, studies from Addis Ababa, Ethiopia, report an incidence of 2.6 per 1000 deliveries [5]. This difference may be due to patient factors such as booking status. Additionally, a study from a university teaching hospital in Nigeria reports an incidence of 6.2 per 1000 deliveries, which is higher than the rate observed at our hospital [22].

In this study, the rate of uterine rupture was 122 out of 162 cases (75.3%). This confirms that uterine rupture remains the leading cause of PH, accounting for 67.8% of cases [9, 23]. Although the incidence of uterine rupture in this study is 75.3%, this figure does not include women whose ruptured uteri were repaired. The high rate may be due to obstetricians’ preference for performing emergency hysterectomies rather than repairs, except in low-parity patients without evidence of sepsis or easily repairable tears [23].

Of the 122 cases of uterine rupture, 23 (18.8%) involved previously scarred uterus. This figure remains worryingly high, as it suggests that not all patients with cesarean scars are referred to hospital-level care. Seven out of 33 cases of previous cesarean deliveries were referred from health centers as cases of uterine rupture. This highlights the need for increased education for women with scarred uteri about the importance of early ANC in subsequent pregnancies. Additionally, even today, early admission at 38 weeks should be offered to women who live far from the hospital, lack transportation, or are known to be poor antenatal attenders. This approach could help further reduce the incidence of uterine rupture.

Perinatal mortality from uterine rupture remains high, with our study reporting a rate of 116 out of 122 cases (95.08%). This is notably higher compared to other studies done by Sebitloane and Bayable [23, 24]. Early presentation to a hospital and a high index of suspicion by healthcare professionals are crucial for improving perinatal salvage rates.

Uncontrolled hemorrhage secondary to uterine atony was the second most common indication for PH, accounting for 17.3% of cases. Other reports have found it to be the leading indication [8, 9, 13]. Most of these cases occurred during cesarean delivery (13 out of 28 cases; 46.42%). Since bleeding typically originates from the placental bed, conservative methods were employed to control bleeding before proceeding to hysterectomy. In some cases, medical methods using various uterotonic agents were utilized (23 out of 28 cases; 82.1%), while surgical methods were used in 2 cases (7.1%), and a combination of both methods was applied in the remaining 3 cases (10.7%). Recent documentation includes stepwise devascularization of the uterus [23]. These surgical steps can be time-consuming, especially in shocked patients, and may result in greater morbidity compared to immediate hysterectomy following the placement of hemostatic sutures and application of pressure to control hemorrhage. Due to the retrospective nature of this study, poor documentation made it challenging to evaluate the procedures performed in detail.

Most studies on EPH have identified morbidly adherent placenta as a frequent indication for the procedure [25]. In our study, it accounted for 8 out of 162 cases (4.9%). This lower incidence may be due to the relative prevalence of uterine rupture and uterine atony in our region. Nonetheless, steps should be taken to exclude morbidly adherent placenta in patients with placenta previa and previously scarred uteri.

The maternal mortality rate in our study was 38 out of 162 cases (23.5%), which is higher than rates reported in other studies [9, 26]. Similarly, the overall perinatal mortality rate of 123 out of 162 cases (75.9%) is also higher than reported elsewhere [9].

Complications from PH are high due to factors such as increased blood supply to the pelvic organs during pregnancy, distorted pelvic anatomy from an enlarged uterus, the unplanned nature of the surgery, and the need to complete the procedure quickly. 62% of our patients developed complications, which is higher than rates reported in other studies. The incidence of complications is influenced by factors including the type of surgery, the indication for surgery, and the use of perioperative antibiotics [23].

Most of our patients underwent total hysterectomy, a procedure recommended by many authors [23]. Other authors have noted long-term issues associated with subtotal hysterectomy, such as vaginal discharge, acyclic bleeding, and the need for cervical cytology. In developing countries where cervicovaginal infections are common and cervical cancer is prevalent, total hysterectomy is often preferred [23].

Maternal blood pressure (< 90/60 mmHg) before surgery is a critical factor influencing the incidence and outcomes of PH as we found in the current study 0.3 (95% CI: 0.1–0.8). High maternal blood pressure can contribute to complications such as uterine atony and hemorrhage, which are critical factors leading to the need for hysterectomy [27–29]. Being transfused with blood before surgery is significantly associated with maternal outcome in the current study 6.8 (95% CI: 1.1–43.4) which is supported by previous reports elsewhere [30–32]. The need for a blood transfusion before surgery often suggests the presence of severe anemia, hemorrhage, or other complications such as placenta previa, placenta accreta, or uterine rupture. Delivery in the study hospital was also the predictor for the maternal outcome 16.1 (95% CI: 2.2–117.7). This finding suggests that the characteristics and quality of care at the study hospital may have an important influence on maternal health outcomes. While the reasons for this association are not directly explored in this study, it is possible that factors such as the expertise of the healthcare providers, the availability of medical resources, and the hospital’s protocols for managing high-risk deliveries could play a role in influencing maternal outcomes. Given the association observed, it is essential for future studies to examine the specific factors within the hospital setting that may contribute to such outcomes. Additionally, these findings may highlight the need for hospital-level interventions to improve maternal care and outcomes, particularly in settings with limited resources.

### Limitation

This study used retrospective design. Lack of document and poor recording of the detailed procedure during surgery limited some of our information. Hence we would approach the generalizability of the study with caution. Additionally, for the predictor variable “delivery in the study site,” the wide 95% confidence interval observed for maternal outcomes (alive/died) can be attributed to the rarity of the event, only 3 deaths occurred in the study hospital. This gap might be bridged with the future studies include a larger sample size.

## Conclusion

The incidence of PH at the study hospital is notably high, with a significant proportion of patients coming from rural areas. Uterine rupture has emerged as the leading cause of PH in our setting. Several key factors significantly associated with PH, including maternal blood pressure prior to surgery, the need for preoperative blood transfusion, a history of cesarean sections, multiple pregnancies, and antepartum hemorrhage. Based on the study’s findings, it is recommended to enhance early detection and management of uterine rupture and hypertensive disorders, particularly through improved antenatal care that monitors blood pressure in high-risk pregnancies. Strengthening blood transfusion services and ensuring adequate ICU resources are crucial for managing severe cases. Infection control measures, and proper surgical techniques should be emphasized to reduce wound infections. There is also a need to standardize hysterectomy versus repair criteria, with an individualized approach. Post-operative care should include intensive monitoring to prevent complications, while focusing on improving fetal and neonatal care to reduce perinatal mortality. Finally, future research with larger sample sizes is necessary to further investigate factors influencing maternal outcomes. These actions aim to improve both maternal and perinatal outcomes.

## Data Availability

The datasets used in this study are available from the corresponding author as per the guideline of the journal up on request.

## Abbreviations

AFE: Amniotic fluid embolism
ANC: Antenatal Care
CS: Cesarean Section
DIC: Disseminated intravascular coagulation
EPH: Emergency Peripartum Hysterectomy
ICU: Intensive care unit
MOH: Minister of Health
OBGYN: Obstetrics and Gynecology
MOF: Multiple organ failure
PH: Peripartum Hysterectomy
SPMMC: St. Paul Hospital millennium medical college
SPSS: Statistical package for social sciences
SVD: Spontaneous Vaginal Delivery
VD: Vaginal delivery
WHO: World Health Organization
WSU: Wolaita Sodo University
WSUCSH: Wolaita Sodo University Comprehensive Specialized Hospital
